# Characterisation of mouse monoclonal antibodies against rhesus macaque killer immunoglobulin-like receptors KIR3D

**DOI:** 10.1007/s00251-012-0640-2

**Published:** 2012-08-15

**Authors:** Meike Hermes, Sandra Weil, Ariane Groth, Ralf Dressel, Joachim Koch, Lutz Walter

**Affiliations:** 1 Primate Genetics Laboratory, German Primate Center, Leibniz Institute for Primate Research, Kellnerweg 4, 37077 Göttingen, Germany; 2 Institute of Biomedical Research, Georg-Speyer-Haus, Frankfurt am Main, Germany; 3 Department of Cellular and Molecular Immunology, University of Göttingen, Göttingen, Germany

**Keywords:** Monoclonal antibodies, Killer immunoglobulin-like receptor (KIR), Epitope mapping, Rhesus macaque

## Abstract

Killer immunoglobulin-like receptors (KIRs) represent a highly polymorphic and diverse gene family in rhesus macaques. Analyses of the respective gene products have been hampered until now due to non-availability of specific monoclonal antibodies and failure of cross-reactivity of anti-human KIR antibodies. We utilised one activating (KIR3DSW08) and two inhibitory (KIR3DLW03 and KIR3DL05) rhesus macaque KIR-Fc fusion proteins for generation of monoclonal antibodies in mice. Besides broadly reacting ones, we obtained anti-rhesus macaque KIR antibodies with intermediate and with single specificity. These monoclonal antibodies were tested for binding to a panel of rhesus macaque KIR proteins after heterologous expression on transiently transfected cells. Epitope mapping identified two polymorphic regions that are located next to each other in the mature KIR proteins. The availability of monoclonal antibodies against rhesus macaque KIR proteins will enable future studies on KIR at the protein level in rhesus macaques as important animal models of human infectious diseases.

**Electronic supplementary material:**

The online version of this article (doi:10.1007/s00251-012-0640-2) contains supplementary material, which is available to authorized users.

There has been substantial progress recently in the analysis of the *KIR* gene family of macaque species since their initial description more than a decade ago (Grendell et al. 2001; Hershberger et al. 2001). Rhesus macaque *KIR* genes and haplotypes turned out to be at least as polymorphic and diverse as their human counterparts (Blokhuis et al. 2011; Kruse et al. 2010; Moreland et al. 2011; Hershberger et al. 2001). Whereas members of all *KIR* lineages known in Old World monkeys and apes/humans are present, a particular expansion of lineage II *KIR*, i.e. *KIR3D* genes, was noticed in rhesus and other macaque species (Bimber et al. 2008; Blokhuis et al. 2010, 2011; Kruse et al. 2010). This expansion of *KIR3D* genes is mirrored by expansion of Mamu-A MHC class I genes (Otting et al. 2005, 2007), which encode ligands for rhesus macaque KIR3D proteins (Colantonio et al. 2011; Rosner et al. 2011). Studies on rhesus macaque KIR proteins have been hampered so far by non-availability of specific monoclonal antibodies (mAbs) and by lack of cross-reactivity of anti-human KIR mAbs. Here, we describe a panel of eight mAbs raised in mice against recombinant rhesus macaque KIR-Fc fusion proteins.

C3H/HeN and C57BL/6 mice were immunised with 100 μg of either KIR3DL05, KIR3DLW03 or KIR3DSW08 recombinant proteins fused to the Fc domain of human IgG1 (Rosner et al. 2011; Older Aguilar et al. 2011). The first immunisation was performed subcutaneously with Titermax Gold (Sigma) as adjuvant, followed by two intra-peritoneal injections at 4 weeks interval. The mice received a final boost by intravenous injection of the KIR-Fc fusion protein without adjuvant. Blood samples were collected before the first and after the third immunisation and serum reactivity was monitored using enzyme-linked immunosorbent assays (ELISA) with the KIR-Fc protein used for immunisation. Generation, selection and cloning of hybridoma cells were performed using the ClonaCell-HY Hybridoma Cloning kit (STEMCELL Technologies) following the manufacturer's protocol and using mouse X63AG8.653 myeloma cell line (German Collection of Microorganisms and Cell Culture, DSMZ). Antibody-secreting hybridoma cells reacting with the KIR-Fc fusion protein but not with control human IgG were selected and cultured in the presence of DMEM/20 % foetal calf serum/1 % penicillin/streptavidin. The immunoglobulin isotypes of the different mAbs were determined with the Pierce Rapid ELISA Mouse mAb Isotyping Kit (Thermo Scientific).

For establishment of *KIR* gene expression constructs, total RNA from peripheral blood mononuclear cells was reverse transcribed using oligo-dT primer and Moloney murine leukaemia virus reverse transcriptase (Promega). As a further source, various *KIR* cDNA clones (Kruse et al. 2010) were used for polymerase chain reaction (PCR) to amplify rhesus macaque *KIR* cDNA with BioTherm Taq DNA Polymerase (Genecraft) using the following primer pairs: KIR-EcoRI-forward I: GATGAATTCAGCACCATGTCGCTCATAG, KIR-EcoRI-forward II: GATGAATTCAGCACCATGTCGCTCATGG, KIR-BamHI-reverse I: GGTGGATCCAGTCTCTTTTTGTCGG and KIR-BamHI-reverse II: GGTGGATCCGGATAGAAGACAACTTTCGATC. PCR products were digested with EcoRI and BamHI and purified and ligated in EcoRI/BamHI-digested pAcGFP-N1 expression vector (Clontech). This vector allows the expression of AcGFP-tagged fusion proteins (Rosner et al. 2010). *KIR*-AcGFP constructs were transiently transfected in HEK293 cells using metafectene according to the manufacturer's guidelines (Biontex). Supernatants of anti-KIR antibody-secreting hybridoma cells were used for staining of KIR-AcGFP-expressing HEK293 cells. Cells (2 × 10^5^) were incubated for 30 min at 4 °C with 50 μL of supernatant and binding was detected with goat anti-mouse IgG-PE-Cy5 polyclonal antibody (SC-3799, Santa Cruz). At least 10,000 AcGFP-positive cells were measured in an LSR II flow cytometer (BD Bioscience) and subsequently analysed with FlowJo 8.8.7 software. The supernatant of antibody-producing hybridoma cells grown in serum-free UltraCHO medium for 3 days was collected, centrifuged (10 min, 200×*g*), filtered (0.45 μm) and finally purified with a protein G sepharose column (GE Healthcare).

Epitope mapping of anti-KIR mAb was performed with peptide spot arrays. Peptides of rhesus macaque KIR3DLW03, KIR3DSW08 or KIR3DL05 were synthesised by Fmoc chemistry at activated PEG spacers on cellulose membranes by automated parallel peptide synthesis on a MultiPep RS instrument (Intavis) as described previously (Dietrich et al. 2012; Koch 2011; Plewnia et al. 2007). Membranes were incubated with 4 μg/ml anti-KIR antibody or anti-mouse IgG/horseradish peroxidase (HRP) (Sigma-Aldrich) as a negative control. Bound primary antibodies were detected with anti-mouse IgG/HRP (Sigma-Aldrich) and chemiluminescent detection with the Super Signal West Femto kit (Thermo Scientific).

Immunisation of mice with rhesus macaque KIR3DL05, KIR3DLW03 and KIR3DSW08 Fc fusion proteins resulted in the establishment of numerous hybridoma clones. Supernatants of the clones were tested in ELISA for binding to respective KIR-Fc proteins that were used for immunisation and against human IgG to identify clones reacting only with the KIR and not the Fc portion. We selected eight clones (Table 1) and determined the specificity of these mAbs for other rhesus macaque KIR proteins using recombinant KIR-Fc proteins in ELISA (not shown) as well as HEK293 cells transiently transfected with different KIR-AcGFP expression constructs (Fig. 1 and Supplemental Figs.1 and 2). Determination of the Ig isotype revealed that all eight mAbs have an IgG1 heavy chain and a kappa light chain (not shown).

**Table 1:** Characterisation of hybridoma clones

Clone	Epitopes recognised in peptide spot arrays	Antigen used for immunisation	Mouse strain used for immunisation
2H9	RCHYRGGFNN	KIR3DLW03	C3H/HeN
	SYPHSPTE		
4H11	RCHYRGGFNN	KIR3DLW03	C3H/HeN
	SYPHSPTE		
5H11	RCHYRGGFNN	KIR3DLW03	C3H/HeN
	SYPHSPTE		
2H5	RCYYRDGLNN	KIR3DL05	C3H/HeN
	SYPHSPTE		
1C7	RCHYRGGFNN	KIR3DSW08	C57BL/6
	SYPHSPTE		
2H3	RCHYRRGLNN	KIR3DSW08	C57BL/6
	SYPHSPTE		
2A4	RCHYRRGLNN	KIR3DSW08	C57BL/6
	SYPHSPTE		
1H4	SYPHSPTE	KIR3DSW08	C57BL/6

Epitope sequences (sequence stretches common in all reactive peptide species) were determined by subtractive alignment of reactive spot sequences from peptide spot arrays

**Fig. 1:**
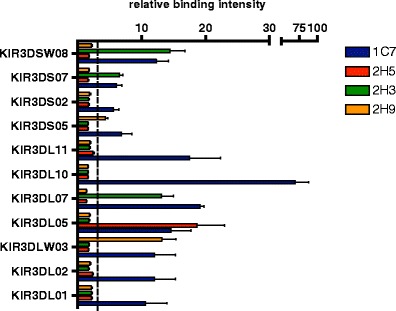
Specificity of binding of mAb to rhesus macaque KIR proteins. Four hybridoma clones are shown: 1C7 reacts broadly and 2H3, 2H5 and 2H9 react specifically. HEK293 cells were transiently transfected with various KIR-AcGFP expression constructs. After gating on AcGFP-positive cells, relative binding intensity was calculated as the ratio of mean fluorescence intensity (MFI) of KIR-AcGFP and MFI of mock-transfected cells. Relative binding intensities above 3 (*threshold line*) were regarded as specific binding. All experiments were carried out at least three times

Clone 1C7 showed broad binding to all tested KIR proteins (Fig. 1), whereas clones 2A4, 1H4, 4H11 (not shown) and 2H3 (Fig. 1) revealed a more restricted reaction pattern. Interestingly, we obtained two clones with high specificity: clone 2H5 reacted only with KIR3DL05 used for immunisation, whereas clone 2H9 reacted strongly with KIR3DLW03 (used for immunisation) and weakly with KIR3DS05 (Fig. 1). In those cases where specific binding is evident, the observed differences between the receptors must not necessarily reflect different avidity of the antibody to the various KIR proteins. Such differences might also be due to (1) differences in transfection efficiency and in amount of transfected DNA, (2) differences in the stability of AcGFP fusion proteins and (3) differences in the stability of inhibitory and activating KIR.

As all mAbs are suitable for immunoblot analysis (not shown), we performed array-based oligo-peptide scanning with spotted 18mer peptides (off-set by three amino acids) covering the entire sequence of several rhesus macaque KIRs (Supplementary Table 1) for epitope identification. The reaction patterns of the tested mAbs were quite similar (Fig. 2a). In particular, peptides PQGGHVTLRCHYRGGFNN (Fig. 2a: spots 7 and 8) and AHAGTYRCRGSYPHSPTG (Fig. 2a: spots 22/23 and 23/24) showed reactivity, indicating that amino acids within these peptides contribute to the epitope. As the 3D structure of human KIR3DL1 was recently published (Vivian et al. 2011), we used this structure to identify the location of the array-positive peptides in the KIR3D three-dimensional structure. Epitope sequences (sequence stretches common in all reactive peptide species) were determined by subtractive alignment of reactive peptide sequences. As can be seen in Fig. 2b, the dominantly reacting peptide stretches RCHYRHRFNN and SHPHSPTG (both sequences from human KIR3L1 in the 3D structure) shown in yellow and red, respectively, are surface exposed and located next to each other in the D0 domain of the KIR3D protein. Therefore, we conclude that the antibodies recognise a conformational epitope. Strikingly, despite the sequence differences of the three KIR3D proteins used for immunisation (Table 1 and Fig. 2c), the mAbs bind to the surface-exposed epitope region in all KIR3D tested. Nevertheless, identical sequences at the epitope are not a guarantee for binding, as for example antibody 2H3 (KIR3DSW08, KIR3DS07 and KIR3DL07) does not bind to KIR3DL05 and KIR3DL10 (Fig. 1), despite both having the same epitope-constituting peptide sequences as KIR3DL07 and KIR3DS07. Thus, we hypothesise that other amino acid differences might contribute to lower (cross-reactive KIRs) the avidity or even abolish binding of the antibody to the conformational epitope.

**Fig. 2:**
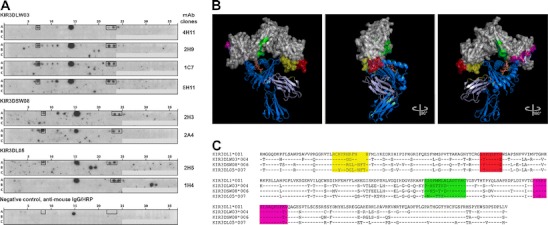
Epitope mapping of anti-KIR mAb. **a** Peptide spot arrays of three rhesus macaque KIR3D proteins (spot sequences, Supplementary Table 1) were incubated with anti-KIR mAbs or anti-mouse IgG/HRP as negative control (one representative array shown). Specific epitopes (*boxes*) were identified in spots A7 and A22/23 (KIR3DL03) and spots A8 and A23/24 (KIR3DSW08; KIR3DL05), respectively. Spots A14 and A15 correspond to a non-specific reactivity, since they are detected in the negative control as well. Additional reactive peptides for mAb 2H5 (spots B18 and B19, marked *green* in **b** and **c**) and 1H4 (spots B31 and B32, marked *magenta* in **b** and **c**) were identified and presumably represent methodical artefacts, e.g. spots corresponding to positions B31 and B32 were also seen for other hybridoma upon overexposure of the blot. **b** Structure of the KIR3DL1*001-pHLA-B*5701 complex (Vivian et al. 2011; PDB accession number 3VH8) with coloured anti-KIR mAb epitopes after subtractive alignment. The KIR3DL1*001 surface is shown in *grey*, the HLA structure in *blue*, β_2_-microglobulin in *light blue* and the HLA-bound peptide (LSSPVTKSF) in *orange*. The identified epitopes from the *spot arrays* are depicted in *yellow* (PRGGHVTLRCHYRHRFNN; spots 7 and 8) and *red* (AHAGNYTCRGSHPHSPTG; spots 22/23 and 23/24). **c** Sequence alignment of the different KIR3D subtypes. Monoclonal antibody epitopes are coloured corresponding to **b**

The mAbs were derived from inbred mouse strains C3H/HeN and C57BL/6, yet the epitopes recognised by the various mAbs are at corresponding positions, suggesting that this position is immunodominant in mice or at least in the two mouse strains used. Indeed, most rhesus macaque KIR inhibitory and activating KIR proteins can be distinguished at these epitopes, with only few allelic polymorphisms (not shown). Thus, despite the fact that we have tested only single alleles of the various KIR proteins (Fig. 1), we expect our specific mAbs to react with most if not all alleles of the respective rhesus macaque KIR proteins. The availability of monoclonal antibodies against rhesus macaque KIR proteins now enables studies on KIR at the protein level in rhesus macaques as animal models of human infectious diseases.

## Electronic supplementary material

Below is the link to the electronic supplementary material.

ESM 1 (PPTX 154 kb)

ESM 2 (PPTX 889 kb)

Supplementary Table 1 Peptide spot array sequences of the three rhesus macaque KIR3D proteins. (DOCX 27 kb)
