# Supplementary material for: Characterisation of mouse monoclonal antibodies against rhesus macaque killer immunoglobulin-like receptors KIR3D
Source: Immunogenetics. 2012 Aug 15;64(11):845–8. doi: 10.1007/s00251-012-0640-2 (PMC3470681; doi:10.1007/s00251-012-0640-2)
Supplement: Supplementary file 1 — (PPTX 154 kb) [file 251_2012_640_MOESM1_ESM.pptx]

## Slide 1
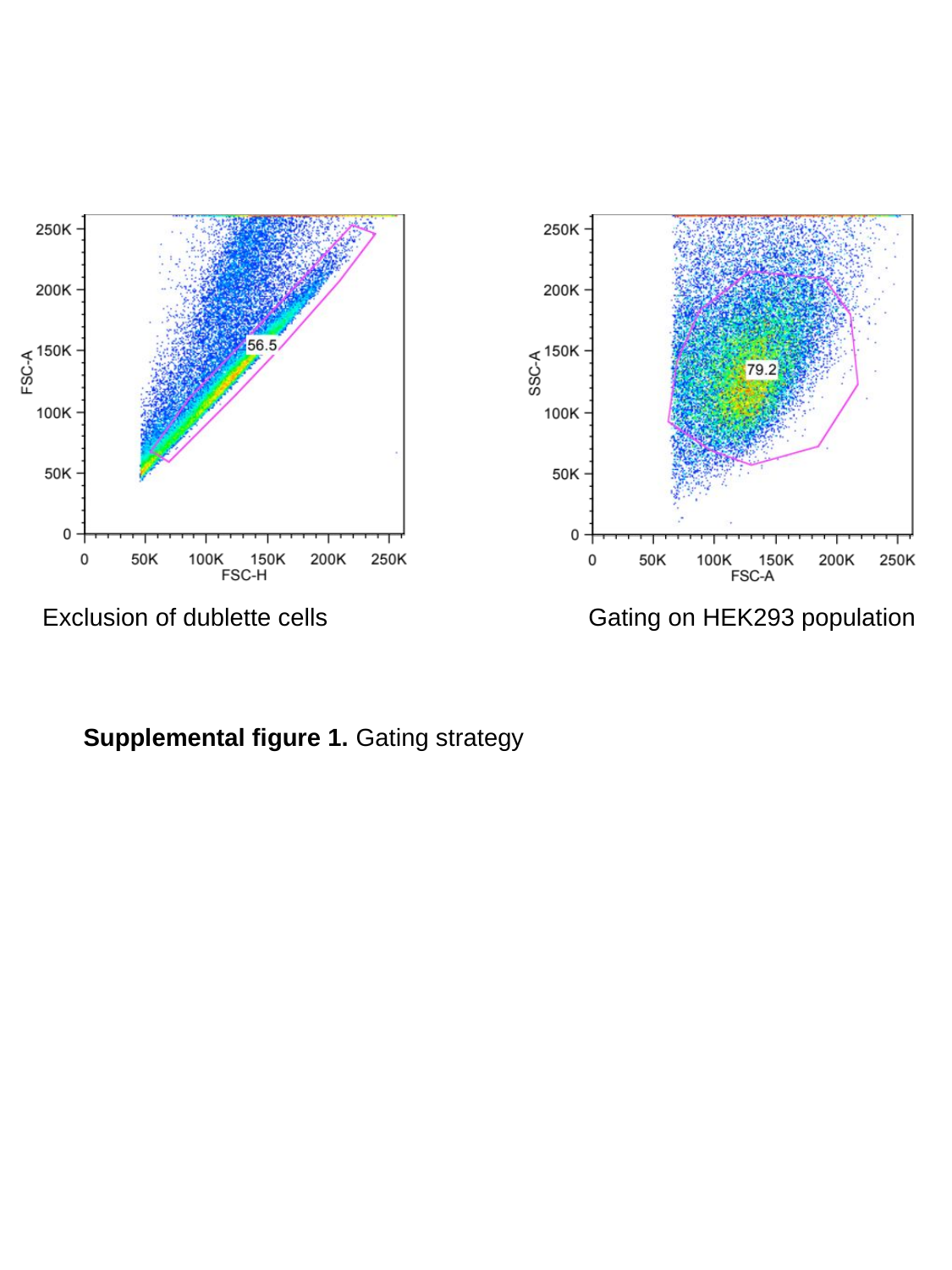

Exclusion of dublette cells
Gating on HEK293 population
Supplemental figure 1. Gating strategy
