# Supplementary material for: Characterisation of mouse monoclonal antibodies against rhesus macaque killer immunoglobulin-like receptors KIR3D
Source: Immunogenetics. 2012 Aug 15;64(11):845–8. doi: 10.1007/s00251-012-0640-2 (PMC3470681; doi:10.1007/s00251-012-0640-2)
Supplement: Supplementary file 2 — (PPTX 889 kb) [file 251_2012_640_MOESM2_ESM.pptx]

## Slide 1
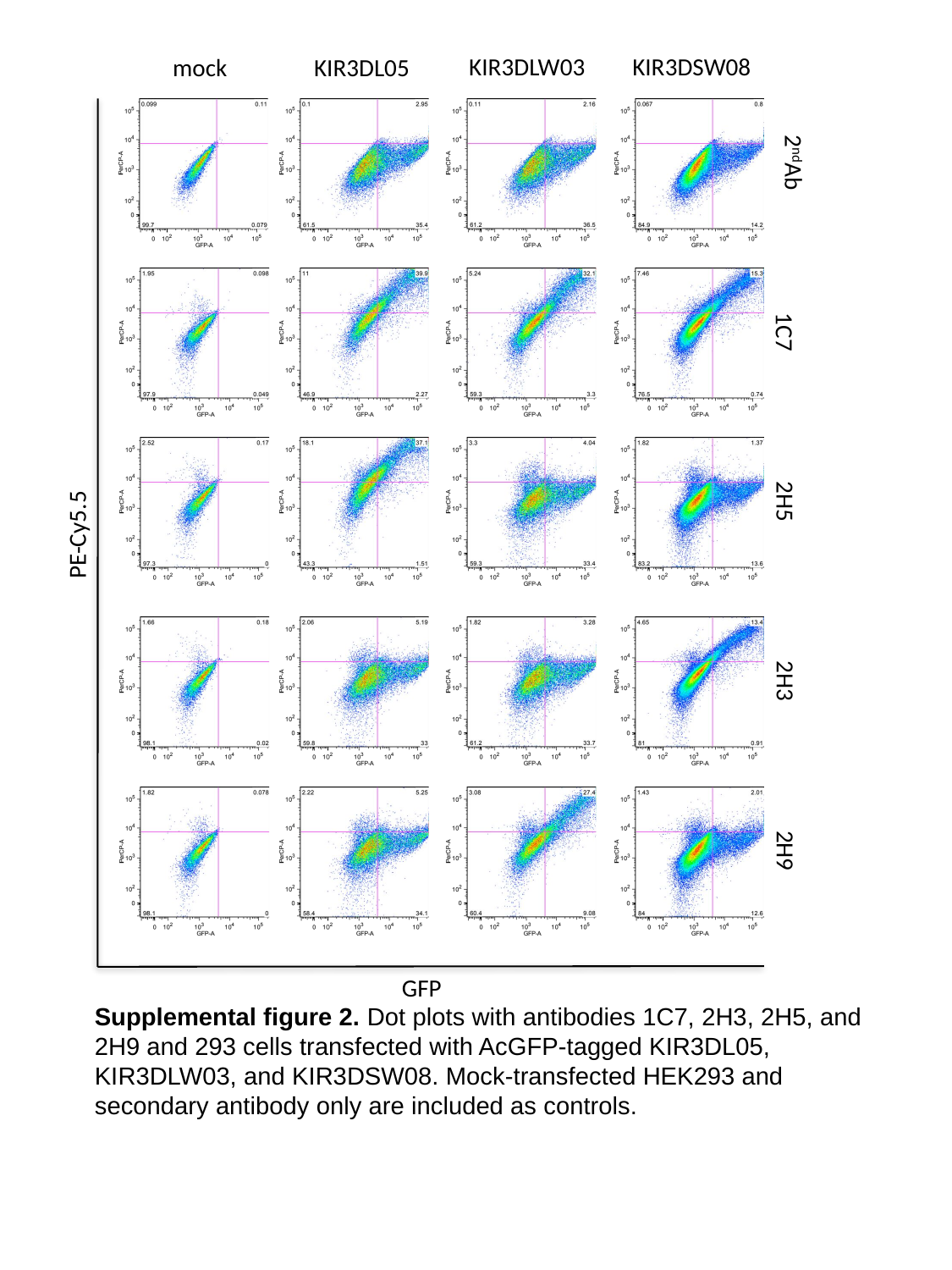

KIR3DSW08
KIR3DLW03
KIR3DL05
mock
2ndAb
1C7
2H5
PE-Cy5.5
2H3
2H9
GFP
Supplemental figure 2. Dot plots with antibodies 1C7, 2H3, 2H5, and 2H9 and 293 cells transfected with AcGFP-tagged KIR3DL05, KIR3DLW03, and KIR3DSW08. Mock-transfected HEK293 and secondary antibody only are included as controls.
