# Supplementary material for: Characterisation of mouse monoclonal antibodies against rhesus macaque killer immunoglobulin-like receptors KIR3D
Source: Immunogenetics. 2012 Aug 15;64(11):845–8. doi: 10.1007/s00251-012-0640-2 (PMC3470681; doi:10.1007/s00251-012-0640-2)
Supplement: Supplementary file 3 — Peptide spot array sequences of the three rhesus macaque KIR3D proteins. (DOCX 27 kb) [file 251_2012_640_MOESM3_ESM.docx]

**Supplementary Table 1:** Peptide spot array sequences of the three rhesus macaque KIR3D proteins.

| **spot** | **KIR3DLW03** | **KIR3DSW08** | **KIR3DL05** |
| --- | --- | --- | --- |
|  |  |  |  |
| A 1  A 2  A 3  A 4  A 5  A 6  A 7  A 8  A 9  A10  A11  A12  A13  A14  A15  A16  A17  A18  A19  A20  A21  A22  A23  A24  A25 A26  A27  A28 A29  A30  A31  A32  A33  A34  A35  A36  B 1  B 2  B 3  B 4  B 5  B 6  B 7  B 8  B 9  B10  B11  B12  B13  B14  B15  B16  B17  B18  B19  B20  B21  B22  B23  B24  B25  B26  B27  B28  B29  B30  B31  B32  B33  B34  B35  B36  C 1  C 2  C 3  C 4  C 5  C 6  C 7  C 8  C 9  C10  C11  C12  C13  C14  C15  C16  C17  C18  C19  C20  C21  C22  C23 | H-T-G-G-Q-D-K-N-F-L-S-A-W-P-S-P-V-V  G-Q-D-K-N-F-L-S-A-W-P-S-P-V-V-P-Q-G  K-N-F-L-S-A-W-P-S-P-V-V-P-Q-G-G-H-V  L-S-A-W-P-S-P-V-V-P-Q-G-G-H-V-T-L-R  W-P-S-P-V-V-P-Q-G-G-H-V-T-L-R-C-H-Y  P-V-V-P-Q-G-G-H-V-T-L-R-C-H-Y-R-G-G  P-Q-G-G-H-V-T-L-R-C-H-Y-R-G-G-F-N-N  G-H-V-T-L-R-C-H-Y-R-G-G-F-N-N-F-T-L  T-L-R-C-H-Y-R-G-G-F-N-N-F-T-L-Y-K-D  C-H-Y-R-G-G-F-N-N-F-T-L-Y-K-D-D-R-S  R-G-G-F-N-N-F-T-L-Y-K-D-D-R-S-H-I-P  F-N-N-F-T-L-Y-K-D-D-R-S-H-I-P-I-F-H  F-T-L-Y-K-D-D-R-S-H-I-P-I-F-H-S-R-I  Y-K-D-D-R-S-H-I-P-I-F-H-S-R-I-F-Q-E  D-R-S-H-I-P-I-F-H-S-R-I-F-Q-E-S-F-L  H-I-P-I-F-H-S-R-I-F-Q-E-S-F-L-M-G-P  I-F-H-S-R-I-F-Q-E-S-F-L-M-G-P-V-T-P  S-R-I-F-Q-E-S-F-L-M-G-P-V-T-P-A-H-A  F-Q-E-S-F-L-M-G-P-V-T-P-A-H-A-G-T-Y  S-F-L-M-G-P-V-T-P-A-H-A-G-T-Y-R-C-R  M-G-P-V-T-P-A-H-A-G-T-Y-R-C-R-G-S-Y  V-T-P-A-H-A-G-T-Y-R-C-R-G-S-Y-P-H-S  A-H-A-G-T-Y-R-C-R-G-S-Y-P-H-S-P-T-E  G-T-Y-R-C-R-G-S-Y-P-H-S-P-T-E-W-S-T  R-C-R-G-S-Y-P-H-S-P-T-E-W-S-T-P-S-N  G-S-Y-P-H-S-P-T-E-W-S-T-P-S-N-P-L-A  P-H-S-P-T-E-W-S-T-P-S-N-P-L-A-I-R-V  P-T-E-W-S-T-P-S-N-P-L-A-I-R-V-T-G-V  W-S-T-P-S-N-P-L-A-I-R-V-T-G-V-H-K-K  P-S-N-P-L-A-I-R-V-T-G-V-H-K-K-P-S-L  P-L-A-I-R-V-T-G-V-H-K-K-P-S-L-L-A-L  I-R-V-T-G-V-H-K-K-P-S-L-L-A-L-P-G-P  T-G-V-H-K-K-P-S-L-L-A-L-P-G-P-L-V-K  H-K-K-P-S-L-L-A-L-P-G-P-L-V-K-S-G-E  P-S-L-L-A-L-P-G-P-L-V-K-S-G-E-T-V-T  L-A-L-P-G-P-L-V-K-S-G-E-T-V-T-L-Q-C  P-G-P-L-V-K-S-G-E-T-V-T-L-Q-C-S-S-D  L-V-K-S-G-E-T-V-T-L-Q-C-S-S-D-T-V-F  S-G-E-T-V-T-L-Q-C-S-S-D-T-V-F-E-H-F  T-V-T-L-Q-C-S-S-D-T-V-F-E-H-F-F-L-H  L-Q-C-S-S-D-T-V-F-E-H-F-F-L-H-R-E-V  S-S-D-T-V-F-E-H-F-F-L-H-R-E-V-T-L-E  T-V-F-E-H-F-F-L-H-R-E-V-T-L-E-E-P-L  E-H-F-F-L-H-R-E-V-T-L-E-E-P-L-H-L-V  F-L-H-R-E-V-T-L-E-E-P-L-H-L-V-G-E-L  R-E-V-T-L-E-E-P-L-H-L-V-G-E-L-H-G-G  T-L-E-E-P-L-H-L-V-G-E-L-H-G-G-G-S-Q  E-P-L-H-L-V-G-E-L-H-G-G-G-S-Q-A-K-Y  H-L-V-G-E-L-H-G-G-G-S-Q-A-K-Y-P-I-N  G-E-L-H-G-G-G-S-Q-A-K-Y-P-I-N-S-T-T  H-G-G-G-S-Q-A-K-Y-P-I-N-S-T-T-S-D-L  G-S-Q-A-K-Y-P-I-N-S-T-T-S-D-L-A-G-T  A-K-Y-P-I-N-S-T-T-S-D-L-A-G-T-Y-R-C  P-I-N-S-T-T-S-D-L-A-G-T-Y-R-C-Y-G-S  S-T-T-S-D-L-A-G-T-Y-R-C-Y-G-S-F-T-H  S-D-L-A-G-T-Y-R-C-Y-G-S-F-T-H-S-P-Y  A-G-T-Y-R-C-Y-G-S-F-T-H-S-P-Y-V-L-S  Y-R-C-Y-G-S-F-T-H-S-P-Y-V-L-S-A-P-S  Y-G-S-F-T-H-S-P-Y-V-L-S-A-P-S-D-P-L  F-T-H-S-P-Y-V-L-S-A-P-S-D-P-L-D-I-V  S-P-Y-V-L-S-A-P-S-D-P-L-D-I-V-I-T-G  V-L-S-A-P-S-D-P-L-D-I-V-I-T-G-L-Y-E  A-P-S-D-P-L-D-I-V-I-T-G-L-Y-E-K-P-S  D-P-L-D-I-V-I-T-G-L-Y-E-K-P-S-L-S-A  D-I-V-I-T-G-L-Y-E-K-P-S-L-S-A-Q-P-G  I-T-G-L-Y-E-K-P-S-L-S-A-Q-P-G-P-T-V  L-Y-E-K-P-S-L-S-A-Q-P-G-P-T-V-Q-A-G  K-P-S-L-S-A-Q-P-G-P-T-V-Q-A-G-E-N-V  L-S-A-Q-P-G-P-T-V-Q-A-G-E-N-V-T-L-S  Q-P-G-P-T-V-Q-A-G-E-N-V-T-L-S-C-S-S  P-T-V-Q-A-G-E-N-V-T-L-S-C-S-S-R-C-S  Q-A-G-E-N-V-T-L-S-C-S-S-R-C-S-F-D-M  E-N-V-T-L-S-C-S-S-R-C-S-F-D-M-Y-H-L  T-L-S-C-S-S-R-C-S-F-D-M-Y-H-L-S-R-E  C-S-S-R-C-S-F-D-M-Y-H-L-S-R-E-G-E-A  R-C-S-F-D-M-Y-H-L-S-R-E-G-E-A-R-E-L  F-D-M-Y-H-L-S-R-E-G-E-A-R-E-L-S-L-S  Y-H-L-S-R-E-G-E-A-R-E-L-S-L-S-A-V-R  S-R-E-G-E-A-R-E-L-S-L-S-A-V-R-S-V-N  G-E-A-R-E-L-S-L-S-A-V-R-S-V-N-G-T-F  R-E-L-S-L-S-A-V-R-S-V-N-G-T-F-Q-G-D  S-L-S-A-V-R-S-V-N-G-T-F-Q-G-D-F-P-L  A-V-R-S-V-N-G-T-F-Q-G-D-F-P-L-G-P-A  S-V-N-G-T-F-Q-G-D-F-P-L-G-P-A-T-H-G  G-T-F-Q-G-D-F-P-L-G-P-A-T-H-G-G-T-Y  Q-G-D-F-P-L-G-P-A-T-H-G-G-T-Y-R-C-F  F-P-L-G-P-A-T-H-G-G-T-Y-R-C-F-G-S-F  G-P-A-T-H-G-G-T-Y-R-C-F-G-S-F-R-T-T  T-H-G-G-T-Y-R-C-F-G-S-F-R-T-T-P-Y-K  G-T-Y-R-C-F-G-S-F-R-T-T-P-Y-K-W-S-D  R-C-F-G-S-F-R-T-T-P-Y-K-W-S-D-P-S-D  G-S-F-R-T-T-P-Y-K-W-S-D-P-S-D-P-L-P  R-T-T-P-Y-K-W-S-D-P-S-D-P-L-P-V-S-V  P-Y-K-W-S-D-P-S-D-P-L-P-V-S-V-T-G-N | H-T-G-G-Q-D-K-T-S-L-S-A-R-P-S-A-V-V  G-Q-D-K-T-S-L-S-A-R-P-S-A-V-V-P-Q-G  K-T-S-L-S-A-R-P-S-A-V-V-P-Q-G-G-H-V  L-S-A-R-P-S-A-V-V-P-Q-G-G-H-V-T-L-R  R-P-S-A-V-V-P-Q-G-G-H-V-T-L-R-C-H-Y  A-V-V-P-Q-G-G-H-V-T-L-R-C-H-Y-R-R-G  P-Q-G-G-H-V-T-L-R-C-H-Y-R-R-G-L-N-N  G-H-V-T-L-R-C-H-Y-R-R-G-L-N-N-F-T-N  T-L-R-C-H-Y-R-R-G-L-N-N-F-T-N-F-T-L  C-H-Y-R-R-G-L-N-N-F-T-N-F-T-L-Y-K-D  R-R-G-L-N-N-F-T-N-F-T-L-Y-K-D-D-R-S  L-N-N-F-T-N-F-T-L-Y-K-D-D-R-S-H-V-P  F-T-N-F-T-L-Y-K-D-D-R-S-H-V-P-V-F-H  F-T-L-Y-K-D-D-R-S-H-V-P-V-F-H-S-R-I  Y-K-D-D-R-S-H-V-P-V-F-H-S-R-I-F-Q-E  D-R-S-H-V-P-V-F-H-S-R-I-F-Q-E-S-F-L  H-V-P-V-F-H-S-R-I-F-Q-E-S-F-L-M-G-P  V-F-H-S-R-I-F-Q-E-S-F-L-M-G-P-V-T-Q  S-R-I-F-Q-E-S-F-L-M-G-P-V-T-Q-A-H-T  F-Q-E-S-F-L-M-G-P-V-T-Q-A-H-T-G-T-Y  S-F-L-M-G-P-V-T-Q-A-H-T-G-T-Y-R-C-R  M-G-P-V-T-Q-A-H-T-G-T-Y-R-C-R-G-S-Y  V-T-Q-A-H-T-G-T-Y-R-C-R-G-S-Y-P-H-S  A-H-T-G-T-Y-R-C-R-G-S-Y-P-H-S-P-T-E  G-T-Y-R-C-R-G-S-Y-P-H-S-P-T-E-W-S-A  R-C-R-G-S-Y-P-H-S-P-T-E-W-S-A-L-S-E  G-S-Y-P-H-S-P-T-E-W-S-A-L-S-E-P-L-A  P-H-S-P-T-E-W-S-A-L-S-E-P-L-A-I-M-V  P-T-E-W-S-A-L-S-E-P-L-A-I-M-V-T-G-V  W-S-A-L-S-E-P-L-A-I-M-V-T-G-V-H-R-K  L-S-E-P-L-A-I-M-V-T-G-V-H-R-K-P-S-L  P-L-A-I-M-V-T-G-V-H-R-K-P-S-L-L-A-L  I-M-V-T-G-V-H-R-K-P-S-L-L-A-L-P-G-P  T-G-V-H-R-K-P-S-L-L-A-L-P-G-P-L-V-K  H-R-K-P-S-L-L-A-L-P-G-P-L-V-K-S-G-E  P-S-L-L-A-L-P-G-P-L-V-K-S-G-E-T-V-T  L-A-L-P-G-P-L-V-K-S-G-E-T-V-T-L-Q-C  P-G-P-L-V-K-S-G-E-T-V-T-L-Q-C-S-S-D  L-V-K-S-G-E-T-V-T-L-Q-C-S-S-D-M-V-F  S-G-E-T-V-T-L-Q-C-S-S-D-M-V-F-E-H-F  T-V-T-L-Q-C-S-S-D-M-V-F-E-H-F-F-L-H  L-Q-C-S-S-D-M-V-F-E-H-F-F-L-H-S-E-V  S-S-D-M-V-F-E-H-F-F-L-H-S-E-V-T-F-E  M-V-F-E-H-F-F-L-H-S-E-V-T-F-E-E-L-L  E-H-F-F-L-H-S-E-V-T-F-E-E-L-L-H-L-V  F-L-H-S-E-V-T-F-E-E-L-L-H-L-V-G-E-L  S-E-V-T-F-E-E-L-L-H-L-V-G-E-L-H-G-G  T-F-E-E-L-L-H-L-V-G-E-L-H-G-G-G-S-Q  E-L-L-H-L-V-G-E-L-H-G-G-G-S-Q-A-N-Y  H-L-V-G-E-L-H-G-G-G-S-Q-A-N-Y-S-I-N  G-E-L-H-G-G-G-S-Q-A-N-Y-S-I-N-S-M-T  H-G-G-G-S-Q-A-N-Y-S-I-N-S-M-T-S-D-L  G-S-Q-A-N-Y-S-I-N-S-M-T-S-D-L-A-G-T  A-N-Y-S-I-N-S-M-T-S-D-L-A-G-T-Y-R-C  S-I-N-S-M-T-S-D-L-A-G-T-Y-R-C-Y-G-S  S-M-T-S-D-L-A-G-T-Y-R-C-Y-G-S-V-T-H  S-D-L-A-G-T-Y-R-C-Y-G-S-V-T-H-S-P-D  A-G-T-Y-R-C-Y-G-S-V-T-H-S-P-D-V-L-S  Y-R-C-Y-G-S-V-T-H-S-P-D-V-L-S-A-P-S  Y-G-S-V-T-H-S-P-D-V-L-S-A-P-S-D-T-L  V-T-H-S-P-D-V-L-S-A-P-S-D-T-L-D-I-V  S-P-D-V-L-S-A-P-S-D-T-L-D-I-V-I-T-G  V-L-S-A-P-S-D-T-L-D-I-V-I-T-G-L-Y-E  A-P-S-D-T-L-D-I-V-I-T-G-L-Y-E-K-P-S  D-T-L-D-I-V-I-T-G-L-Y-E-K-P-S-L-S-A  D-I-V-I-T-G-L-Y-E-K-P-S-L-S-A-Q-P-G  I-T-G-L-Y-E-K-P-S-L-S-A-Q-P-G-P-T-V  L-Y-E-K-P-S-L-S-A-Q-P-G-P-T-V-Q-A-G  K-P-S-L-S-A-Q-P-G-P-T-V-Q-A-G-E-N-V  L-S-A-Q-P-G-P-T-V-Q-A-G-E-N-V-T-L-S  Q-P-G-P-T-V-Q-A-G-E-N-V-T-L-S-C-S-S  P-T-V-Q-A-G-E-N-V-T-L-S-C-S-S-Q-N-S  Q-A-G-E-N-V-T-L-S-C-S-S-Q-N-S-F-D-M  E-N-V-T-L-S-C-S-S-Q-N-S-F-D-M-Y-H-L  T-L-S-C-S-S-Q-N-S-F-D-M-Y-H-L-S-R-E  C-S-S-Q-N-S-F-D-M-Y-H-L-S-R-E-G-E-A  Q-N-S-F-D-M-Y-H-L-S-R-E-G-E-A-R-G-L  F-D-M-Y-H-L-S-R-E-G-E-A-R-G-L-S-L-S  Y-H-L-S-R-E-G-E-A-R-G-L-S-L-S-A-V-Q  S-R-E-G-E-A-R-G-L-S-L-S-A-V-Q-S-V-N  G-E-A-R-G-L-S-L-S-A-V-Q-S-V-N-R-T-F  R-G-L-S-L-S-A-V-Q-S-V-N-R-T-F-Q-A-D  S-L-S-A-V-Q-S-V-N-R-T-F-Q-A-D-F-P-L  A-V-Q-S-V-N-R-T-F-Q-A-D-F-P-L-G-P-A  S-V-N-R-T-F-Q-A-D-F-P-L-G-P-A-T-H-G  R-T-F-Q-A-D-F-P-L-G-P-A-T-H-G-G-T-Y  Q-A-D-F-P-L-G-P-A-T-H-G-G-T-Y-R-C-F  F-P-L-G-P-A-T-H-G-G-T-Y-R-C-F-G-S-F  G-P-A-T-H-G-G-T-Y-R-C-F-G-S-F-R-T-A  T-H-G-G-T-Y-R-C-F-G-S-F-R-T-A-P-Y-Q  G-T-Y-R-C-F-G-S-F-R-T-A-P-Y-Q-W-S-D  R-C-F-G-S-F-R-T-A-P-Y-Q-W-S-D-P-S-D  G-S-F-R-T-A-P-Y-Q-W-S-D-P-S-D-P-L-S  R-T-A-P-Y-Q-W-S-D-P-S-D-P-L-S-V-S-V  P-Y-Q-W-S-D-P-S-D-P-L-S-V-S-V-T-G-N | H-T-G-G-Q-D-K-I-F-L-S-A-R-P-S-A-V-V  G-Q-D-K-I-F-L-S-A-R-P-S-A-V-V-P-Q-G  K-I-F-L-S-A-R-P-S-A-V-V-P-Q-G-G-H-V  L-S-A-R-P-S-A-V-V-P-Q-G-G-H-V-T-L-R  R-P-S-A-V-V-P-Q-G-G-H-V-T-L-R-C-Y-Y  A-V-V-P-Q-G-G-H-V-T-L-R-C-Y-Y-R-D-G  P-Q-G-G-H-V-T-L-R-C-Y-Y-R-D-G-L-N-N  G-H-V-T-L-R-C-Y-Y-R-D-G-L-N-N-F-T-N  T-L-R-C-Y-Y-R-D-G-L-N-N-F-T-N-F-T-L  C-Y-Y-R-D-G-L-N-N-F-T-N-F-T-L-Y-K-D  R-D-G-L-N-N-F-T-N-F-T-L-Y-K-D-D-R-S  L-N-N-F-T-N-F-T-L-Y-K-D-D-R-S-H-V-P  F-T-N-F-T-L-Y-K-D-D-R-S-H-V-P-I-F-H  F-T-L-Y-K-D-D-R-S-H-V-P-I-F-H-S-R-I  Y-K-D-D-R-S-H-V-P-I-F-H-S-R-I-F-Q-E  D-R-S-H-V-P-I-F-H-S-R-I-F-Q-E-S-F-L  H-V-P-I-F-H-S-R-I-F-Q-E-S-F-L-M-G-P  I-F-H-S-R-I-F-Q-E-S-F-L-M-G-P-V-T-P  S-R-I-F-Q-E-S-F-L-M-G-P-V-T-P-A-H-A  F-Q-E-S-F-L-M-G-P-V-T-P-A-H-A-G-T-Y  S-F-L-M-G-P-V-T-P-A-H-A-G-T-Y-R-C-R  M-G-P-V-T-P-A-H-A-G-T-Y-R-C-R-G-S-Y  V-T-P-A-H-A-G-T-Y-R-C-R-G-S-Y-P-H-S  A-H-A-G-T-Y-R-C-R-G-S-Y-P-H-S-P-T-E  G-T-Y-R-C-R-G-S-Y-P-H-S-P-T-E-W-S-A  R-C-R-G-S-Y-P-H-S-P-T-E-W-S-A-L-S-D  G-S-Y-P-H-S-P-T-E-W-S-A-L-S-D-P-L-A  P-H-S-P-T-E-W-S-A-L-S-D-P-L-A-I-R-V  P-T-E-W-S-A-L-S-D-P-L-A-I-R-V-T-G-V  W-S-A-L-S-D-P-L-A-I-R-V-T-G-V-H-R-K  L-S-D-P-L-A-I-R-V-T-G-V-H-R-K-P-S-L  P-L-A-I-R-V-T-G-V-H-R-K-P-S-L-L-A-L  I-R-V-T-G-V-H-R-K-P-S-L-L-A-L-P-G-P  T-G-V-H-R-K-P-S-L-L-A-L-P-G-P-L-V-K  H-R-K-P-S-L-L-A-L-P-G-P-L-V-K-S-G-E  P-S-L-L-A-L-P-G-P-L-V-K-S-G-E-T-V-T  L-A-L-P-G-P-L-V-K-S-G-E-T-V-T-L-Q-C  P-G-P-L-V-K-S-G-E-T-V-T-L-Q-C-S-S-D  L-V-K-S-G-E-T-V-T-L-Q-C-S-S-D-T-V-F  S-G-E-T-V-T-L-Q-C-S-S-D-T-V-F-E-H-F  T-V-T-L-Q-C-S-S-D-T-V-F-E-H-F-F-L-Q  L-Q-C-S-S-D-T-V-F-E-H-F-F-L-Q-S-E-V  S-S-D-T-V-F-E-H-F-F-L-Q-S-E-V-T-F-K  T-V-F-E-H-F-F-L-Q-S-E-V-T-F-K-K-S-V  E-H-F-F-L-Q-S-E-V-T-F-K-K-S-V-H-L-V  F-L-Q-S-E-V-T-F-K-K-S-V-H-L-V-G-E-L  S-E-V-T-F-K-K-S-V-H-L-V-G-E-L-H-G-G  T-F-K-K-S-V-H-L-V-G-E-L-H-G-G-G-S-Q  K-S-V-H-L-V-G-E-L-H-G-G-G-S-Q-A-N-Y  H-L-V-G-E-L-H-G-G-G-S-Q-A-N-Y-S-M-G  G-E-L-H-G-G-G-S-Q-A-N-Y-S-M-G-P-T-T  H-G-G-G-S-Q-A-N-Y-S-M-G-P-T-T-S-A-L  G-S-Q-A-N-Y-S-M-G-P-T-T-S-A-L-A-G-T  A-N-Y-S-M-G-P-T-T-S-A-L-A-G-T-Y-R-C  S-M-G-P-T-T-S-A-L-A-G-T-Y-R-C-Y-G-S  P-T-T-S-A-L-A-G-T-Y-R-C-Y-G-S-V-T-H  S-A-L-A-G-T-Y-R-C-Y-G-S-V-T-H-S-P-Y  A-G-T-Y-R-C-Y-G-S-V-T-H-S-P-Y-V-L-S  Y-R-C-Y-G-S-V-T-H-S-P-Y-V-L-S-A-P-S  Y-G-S-V-T-H-S-P-Y-V-L-S-A-P-S-D-P-L  V-T-H-S-P-Y-V-L-S-A-P-S-D-P-L-D-I-V  S-P-Y-V-L-S-A-P-S-D-P-L-D-I-V-I-T-G  V-L-S-A-P-S-D-P-L-D-I-V-I-T-G-I-Y-K  A-P-S-D-P-L-D-I-V-I-T-G-I-Y-K-K-P-S  D-P-L-D-I-V-I-T-G-I-Y-K-K-P-S-L-S-A  D-I-V-I-T-G-I-Y-K-K-P-S-L-S-A-Q-P-G  I-T-G-I-Y-K-K-P-S-L-S-A-Q-P-G-P-T-V  I-Y-K-K-P-S-L-S-A-Q-P-G-P-T-V-Q-A-G  K-P-S-L-S-A-Q-P-G-P-T-V-Q-A-G-E-N-V  L-S-A-Q-P-G-P-T-V-Q-A-G-E-N-V-T-L-S  Q-P-G-P-T-V-Q-A-G-E-N-V-T-L-S-C-S-S  P-T-V-Q-A-G-E-N-V-T-L-S-C-S-S-R-R-S  Q-A-G-E-N-V-T-L-S-C-S-S-R-R-S-F-D-M  E-N-V-T-L-S-C-S-S-R-R-S-F-D-M-Y-H-L  T-L-S-C-S-S-R-R-S-F-D-M-Y-H-L-S-R-E  C-S-S-R-R-S-F-D-M-Y-H-L-S-R-E-G-E-T  R-R-S-F-D-M-Y-H-L-S-R-E-G-E-T-H-E-L  F-D-M-Y-H-L-S-R-E-G-E-T-H-E-L-R-L-P  Y-H-L-S-R-E-G-E-T-H-E-L-R-L-P-A-V-P  S-R-E-G-E-T-H-E-L-R-L-P-A-V-P-S-V-N  G-E-T-H-E-L-R-L-P-A-V-P-S-V-N-G-T-F  H-E-L-R-L-P-A-V-P-S-V-N-G-T-F-Q-A-D  R-L-P-A-V-P-S-V-N-G-T-F-Q-A-D-F-P-L  A-V-P-S-V-N-G-T-F-Q-A-D-F-P-L-G-P-V  S-V-N-G-T-F-Q-A-D-F-P-L-G-P-V-T-H-G  G-T-F-Q-A-D-F-P-L-G-P-V-T-H-G-G-T-Y  Q-A-D-F-P-L-G-P-V-T-H-G-G-T-Y-R-C-F  F-P-L-G-P-V-T-H-G-G-T-Y-R-C-F-A-S-F  G-P-V-T-H-G-G-T-Y-R-C-F-A-S-F-R-T-A  T-H-G-G-T-Y-R-C-F-A-S-F-R-T-A-P-Y-E  G-T-Y-R-C-F-A-S-F-R-T-A-P-Y-E-W-S-V  R-C-F-A-S-F-R-T-A-P-Y-E-W-S-V-P-S-D  A-S-F-R-T-A-P-Y-E-W-S-V-P-S-D-P-L-H  R-T-A-P-Y-E-W-S-V-P-S-D-P-L-H-V-S-I  P-Y-E-W-S-V-P-S-D-P-L-H-V-S-I-T-G-N |
